# Quantitative Assessment of Endoscope Tip Angulation Performance in New Endoscopes: A Cross-Sectional Quality Improvement Study

**DOI:** 10.1055/a-2877-9452

**Published:** 2026-06-10

**Authors:** Saksham Kohli, Vikram Kotwal, Mihir Shah, Daksh Ahluwalia, Eric Zhao, Shalini Atluri, Seema Gandhi

**Affiliations:** 1 Department of Internal Medicine 25430 John H Stroger Jr Hospital of Cook CountyChicagoIllinoisUnited States; 2 Division of Gastroenterology and Hepatology 25430 John H Stroger Jr Hospital of Cook CountyChicagoIllinoisUnited States; 3 Medical Student 2468 Rush University Medical CenterChicagoIllinoisUnited States

**Keywords:** quality and logistical aspects, quality management, training, other focus (of reviewers), statistics, performance and complications

## Abstract

**Background** Endoscope tip angulation is a key determinant of procedural maneuverability, yet routine quantitative assessment is not standardized, and data are limited on how quickly new endoscopes fall below manufacturer-specified performance benchmarks during routine use. The aim of this study was to evaluate tip angulation performance in new, apparently functional endoscopes after a period of clinical use and to characterize the prevalence and pattern of below-tolerance findings by scope type.

**Methods** We performed a quality improvement project to evaluate the tip angulation of newly issued endoscopes within nine months of clinical use. In phase 1, endoscopes were systematically assessed by three trained physicians using a reference chart. In phase 2, a company representative independently evaluated endoscopes using a calibrated instrument.

**Results** In all, 32 new endoscopes (18 colonoscopes and 14 gastroscopes) were evaluated. All scopes were used for approximately 9 months. The mean procedure count for colonoscopes was 185.6 (±12.5, range 162–201) and for gastroscopes was 150.6 (±12.0, range 133–166). Totally, 5 out of 18 colonoscopes (27.8%) fell below manufacturer tolerance thresholds for maximal tip deflection in at least one direction. All 14 gastroscopes met manufacturer tolerance thresholds in all four directions.

**Conclusions** This cross-sectional study found that a notable proportion of new colonoscopes fell below manufacturer tolerance thresholds after approximately nine months of clinical use, despite being considered apparently functional. These findings highlight the need for periodic, objective assessment of tip angulation, even in endoscopes without apparent defects. Longitudinal studies are needed to establish deterioration trajectories and inform maintenance guidelines.

## Introduction

Despite the operational importance of optimal endoscope performance, maintenance practices are commonly only reactive, initiated only after overt malfunction, image degradation, or device failure. This approach permits subtle performance degradation, such as reduced tip deflection, to persist for extended periods of time. Even minor changes in tip angulation may potentially increase procedural difficulty and elevate the ergonomic demands placed on the operator. When tip angulation is suboptimal, it is plausible that endoscopists may compensate by applying greater thumb force, increased torque, and exaggerated wrist deviation to achieve visualization and therapeutic positioning, although this has not been directly demonstrated. From a systems perspective, waiting until a scope malfunctions may potentially increase repair costs, as delayed identification of early mechanical wear can convert smaller, targeted servicing needs into more complex and expensive repairs.

Rozeboom et al. performed an evaluation of tip angulation in a similar clinical setting and found that their tested endoscopes did not achieve the manufacturer-specified maximum tip angulation. 1 However, this group did not study how quickly new or newly adjusted endoscopes deviate from optimal angulation with routine use. This group also did not define utilization-based thresholds that can inform standardized screening and proactive servicing.

To address this gap, we performed a cross-sectional quantitative assessment of both gastroscope and colonoscope tip angulation in new endoscopes after approximately nine months of clinical use, characterizing the prevalence and pattern of below-tolerance findings by scope type.

## Methods

This prospective quality improvement study evaluated brand-new colonoscopes and gastroscopes at John H. Stroger, Jr. Hospital of Cook County, with waiver of informed consent. Endoscopes were systematically assessed using a two-phase evaluation protocol: initial screening by trained physician reviewers followed by verification by a company representative using calibrated manufacturer instruments.

### Phase 1: Independent Reviewer Assessment

Twenty endoscopes (10 colonoscopes, 10 gastroscopes) underwent Phase 1 assessment by three trained physician reviewers: S.K. (R1), M.S. (R2), and D.A. (R3), blinded to usage data, who independently evaluated tip deflection in four directions (up, down, left, right) using the company reference chart, yielding 240 individual assessments. Procedure counts were independently recorded by staff not involved in angulation assessments.

### Phase 2: Manufacturer Representative Verification

In Phase 2, the company representative was invited to independently evaluate the endoscopes using calibrated manufacturer instruments. In addition to the 20 primary study scopes, the representative evaluated all other endoscopes available at the institution on the day of assessment (8 additional colonoscopes and 4 additional gastroscopes), for a total of 18 colonoscopes and 14 gastroscopes (32 endoscopes total). Procedure count data were not available for these additional scopes, as they had not been prospectively tracked as part of the primary study cohort. However, all 32 endoscopes were new, issued at the same time, and had been in clinical use for the same approximately nine-month period. Manufacturer tolerance thresholds were: colonoscopes: upward/downward ≥160°, rightward/leftward ≥140°; gastroscopes: upward ≥180°, downward ≥70°, rightward/leftward ≥80°. Endoscopes with any directional measurement below threshold were classified as below tolerance. Inter-rater reliability was assessed using Fleiss’ kappa; statistical significance was defined as *p* < 0.05.

## Results

### Study Population and Scope Characteristics

Thirty-two endoscopes were evaluated: 18 colonoscopes and 14 gastroscopes, out of which, 20 endoscopes (10 colonoscopes and 10 gastroscopes) constituted the primary study cohort that was first assessed by the three trained reviewers. Mean procedure counts for the 20 primary scopes were 185.6 (±12.5, range 162–201) for colonoscopes and 150.6 (±12.0, range 133–166) for gastroscopes, all in clinical use for approximately 9 months.

### Inter-rater Reliability

Inter-rater agreement was excellent: overall Fleiss’ kappa 0.892 (95% CI: 0.847–0.937, *p* < 0.001), with 94.2% overall agreement. Directional kappa values were: up 0.886, down 0.901, left 0.873, right 0.908 (all *p* < 0.001), with percent agreement ranging from 93.3% to 96.7%. These findings validate the reproducibility and consistency of the angulation assessment methodology.

### Phase 1: Reviewer Evaluation

Phase 1 reviewer assessment identified 3 colonoscopes (30%) and 1 gastroscope (10%) as having suboptimal angulation, flagging 4 of 20 scopes (20%) overall.

### Phase 2: Colonoscope Angulation Performance

Phase 2 manufacturer evaluation confirmed 5 of 18 colonoscopes (27.8%) below tolerance in at least one direction ( Table 1 and Fig. 1A ). Among the 10 primary colonoscopes, 3 (30%) were below tolerance, consistent with reviewer findings. All five below-tolerance colonoscopes demonstrated downward angulation below 160°; one additionally fell below the rightward threshold (130°), as illustrated in Fig. 1B . Several within-tolerance colonoscopes clustered at the lower boundary, with 6 measuring exactly 160° downward and 2 measuring exactly 140° rightward. Among the 10 primary colonoscopes with available procedure counts, the three below-tolerance scopes had a numerically higher mean procedure count (194.7) than the seven within-tolerance scopes (181.7), although the small sample size precludes formal statistical comparison.

**Table 1:** Manufacturer representative angulation measurements for 18 colonoscopes. Values represent tip angulation in degrees measured using calibrated instruments. Tolerance thresholds: up ≥160°, down ≥160°, right ≥140°, left ≥140°. Values in **bold** indicate measurements below the manufacturer’s tolerance threshold. – = not available (additional scopes evaluated by representative only). Primary cohort scopes ( *n* = 10) were first assessed by three trained reviewers; additional scopes ( *n* = 8) were evaluated by the manufacturer representative alone.

#	Procedure count	Up (°)	Down (°)	Right (°)	Left (°)	Tolerance threshold status	Cohort
		**≥160**	**≥160**	**≥140**	**≥140**		
1	185	180°	**150°**	**130°**	140°	**Below**	Primary
2	201	180°	**140°**	150°	150°	**Below**	Primary
3	198	160°	**150°**	150°	150°	**Below**	Primary
4	–	170°	**150°**	150°	150°	**Below**	*Additional*
5	–	160°	**150°**	140°	140°	**Below**	*Additional*
6	189	180°	160°	150°	150°	Within	Primary
7	172	180°	160°	150°	160°	Within	Primary
8	162	170°	180°	160°	160°	Within	Primary
9	189	180°	160°	160°	160°	Within	Primary
10	174	180°	170°	150°	150°	Within	Primary
11	193	180°	160°	140°	150°	Within	Primary
12	193	180°	180°	160°	160°	Within	Primary
13	–	180°	160°	150°	160°	Within	*Additional*
14	–	180°	170°	150°	150°	Within	*Additional*
15	–	175°	165°	160°	160°	Within	*Additional*
16	–	180°	170°	160°	160°	Within	*Additional*
17	–	170°	160°	140°	140°	Within	*Additional*
18	–	180°	175°	160°	160°	Within	*Additional*

**Fig. 1:**
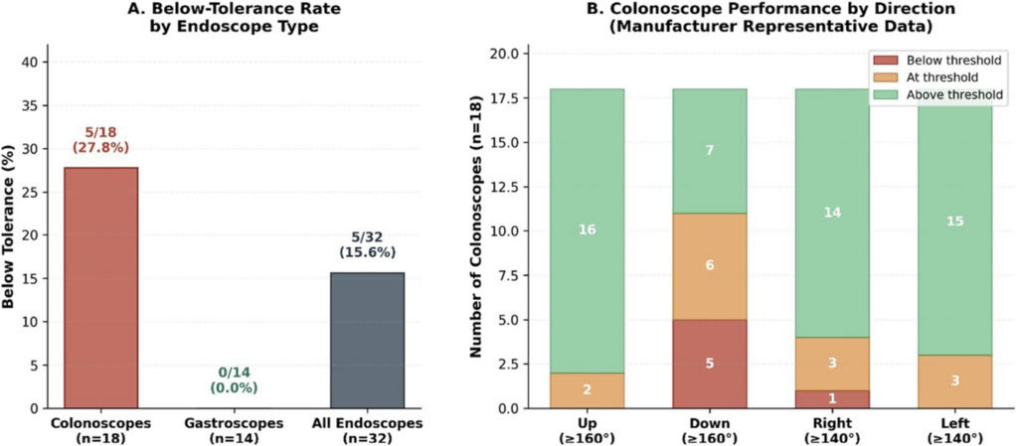
Summary of endoscope angulation performance by scope type and direction. ( **A** ) Below-tolerance rate by endoscope type. Colonoscopes demonstrated a 27.8% below-tolerance rate (5/18) compared to 0% for gastroscopes (0/14), yielding a combined rate of 15.6% (5/32). All below-tolerance findings were confined to colonoscopes. ( **B** ) Colonoscope directional performance based on manufacturer representative measurements ( *n* = 18). Stacked bars show the number of colonoscopes below threshold (red), at threshold (orange), and above threshold (green) for each direction. Downward angulation demonstrated the poorest performance, with 5 scopes below and 6 at the 160° threshold (61.1% at or below threshold). Upward angulation was best preserved, with no scopes below and only 2 at the 160° threshold.

### Phase 2: Gastroscope Angulation Performance

In contrast, all 14 gastroscopes (100%) met tolerance thresholds in all four directions ( Table 2 and Fig. 1A ). One reviewer-flagged gastroscope measured 80° downward by the representative, above the 70° threshold, suggesting reviewer assessment was more conservative than calibrated measurement.

**Table 2:** Manufacturer representative angulation measurements for 14 gastroscopes. Values represent tip angulation in degrees measured using calibrated instruments. Tolerance thresholds: up ≥180°, down ≥70°, right ≥80°, left ≥80°. Values in **bold** indicate measurements at the exact tolerance threshold. All 14 gastroscopes met tolerance thresholds in all four directions. – = not available. Primary cohort scopes ( *n* = 10) were first assessed by three trained reviewers; additional scopes ( *n* = 4) were evaluated by the manufacturer representative alone.

#	Procedure count	Up (°)	Down (°)	Right (°)	Left (°)	Tolerance threshold status	Cohort
		**≥180**	**≥70**	**≥80**	**≥80**		
1	156	185°	80°	90°	95°	Within	Primary
2	137	185°	85°	90°	95°	Within	Primary
3	134	190°	90°	90°	100°	Within	Primary
4	149	190°	80°	90°	100°	Within	Primary
5	157	200°	85°	100°	100°	Within	Primary
6	163	185°	85°	90°	95°	Within	Primary
7	166	185°	85°	95°	90°	Within	Primary
8	157	200°	80°	100°	100°	Within	Primary
9	133	190°	80°	100°	**80°**	Within	Primary
10	154	190°	80°	100°	100°	Within	Primary
11	–	185°	**70°**	90°	90°	Within	*Additional*
12	–	190°	**70°**	90°	**80°**	Within	*Additional*
13	–	190°	90°	100°	90°	Within	*Additional*
14	–	200°	90°	90°	100°	Within	*Additional*

### Combined Analysis by Scope Type

Among 32 tested endoscopes, 5 fell below manufacturer tolerance thresholds, all of which were colonoscopes ( Fig. 1A ). While a formal comparison between scope types is limited by differing tolerance thresholds, this pattern may reflect the greater mechanical demands of colonoscopy, including the forces involved in colonic navigation, loop reduction, and retroflexion maneuvers.

## Discussion

This quality improvement study provides cross-sectional evidence that a notable proportion of new colonoscopes fell below manufacturer tolerance thresholds after approximately nine months of clinical use. We found that 5 of 18 colonoscopes (28%) were below manufacturer tolerance thresholds at the time of assessment. Among the 10 primary colonoscopes with known procedure counts, three were below tolerance, all with counts under 202 procedures. Several additional scopes within the tolerance range demonstrated measurements at or near the lower boundary, with six scopes measuring exactly 160° for downward angulation (the minimum acceptable value). This clustering at the tolerance threshold suggests that further deterioration with continued use could result in a substantially higher proportion of scopes falling below acceptable performance levels in the near term.

Our findings align with and extend the limited existing literature on endoscope mechanical deterioration. A Dutch multicenter study by Rozeboom et al. examining 20 colonoscopes and 5 gastroscopes similarly found that only 10% of colonoscopes achieved maximal tip angulation specified by manufacturers, despite some of the scopes having undergone maintenance checks within one month of evaluation. 1 Notably, their study found that recent maintenance did not significantly improve tip responses, paralleling our observation that deterioration can occur even in relatively new equipment.

The possible relationship between suboptimal scope performance and endoscopist injury represents another important yet underexplored area of concern. Endoscopy-related musculoskeletal injuries (ERIs) affect 39–89% of practicing endoscopists, with hands and fingers/thumb being most commonly affected. 2 A recent systematic review reported an increasing prevalence of ERI in recent years (57% before 2015 vs. 71% after 2015). 3

ERIs are also common among GI fellows, with up to 47% having ERI, often within the first year of fellowship. 4 Studies examining force thresholds during colonoscopy demonstrate that risk thresholds for forces on the left thumb and wrists are exceeded during all colonoscopy subtasks. 5 6 When colonoscope angulation cables stretch or lose optimal tension, endoscopists may need to apply greater pinch force and more extreme wrist deviation to achieve the desired tip deflection, although this mechanistic link has not yet been established. While this study did not measure ergonomic outcomes, the potential link between suboptimal scope performance and increased injury risk warrants investigation. Similarly, the possibility that reduced tip angulation could contribute to incomplete procedures or missed lesions, as suggested by Rozeboom et al., remains to be studied. 1

Current endoscope reprocessing guidelines from ASGE focus almost exclusively on infection control through high-level disinfection protocols, with minimal attention to mechanical performance monitoring. The multisociety guideline on reprocessing flexible GI endoscopes emphasizes visual inspection for damage but provides no specific recommendations for quantitative assessment of tip angulation or cable tension. 7 8 Our findings suggest several potential modifications to current endoscope maintenance practices that warrant further evaluation. First, standardized tip angulation testing should be incorporated into routine endoscope inspection protocols. Using manufacturer-provided reference charts, trained personnel can perform initial screening of angulation performance with high inter-rater reliability, as demonstrated by our three-reviewer protocol. When screening identifies potential deficiencies, manufacturer representative evaluation with calibrated instruments can provide precise quantitative confirmation. Second, the observation that almost 30% of scopes fell below tolerance thresholds and several others clustered at the threshold boundary after fewer than 200 procedures suggests that assessment at an earlier interval than the current annual or semiannual schedules may be warranted. The specific threshold at which screening should occur remains to be determined, and our data do not statistically support a defined cutoff. However, the observation that below-tolerance scopes in our study had procedure counts in the range of 162–201 raises the hypothesis that evaluation at approximately 125–150 procedures could serve as a starting point, a premise that requires validation through longitudinal studies with serial assessments. Third, systematic documentation of angulation performance over scope lifetime would enable institutions to identify scopes at high risk for rapid deterioration, establish institution-specific maintenance thresholds, optimize scope rotation to distribute utilization, and support cost–benefit analyses of early intervention versus replacement.

Several limitations warrant consideration in interpreting our findings. Our cohort consisted of 32 endoscopes, all at one institution and manufactured by a single company which limits generalizability. Larger multi-institutional studies would strengthen confidence in our findings and enable more refined threshold determinations. Because of the cross-sectional design of the study, with scopes being assessed at a single timepoint, we cannot determine the rate of deterioration or establish a causal relationship between procedure volume and angulation loss. Longitudinal studies following individual scopes from first use through end-of-life would provide valuable natural history data. We did not stratify by procedure complexity, patient characteristics, or specific indications, and technically challenging procedures may accelerate deterioration. Multiple endoscopists used these scopes, each with different technique, force application, and attention to equipment handling, and these operator-related factors could not be analyzed. Finally, we did not correlate angulation performance with procedural completion rates, patient comfort, or endoscopist-reported difficulty, and such correlations would strengthen the clinical relevance of our findings.

To conclude, this cross-sectional study found that 5 of 18 colonoscopes (27.8%) were below manufacturer tolerance thresholds after approximately nine months of clinical use, while all 14 gastroscopes met thresholds. These findings support the consideration of routine, standardized angulation assessment as part of endoscope-maintenance protocols. Further research should focus on longitudinal studies establishing deterioration trajectories for both scope types, intervention trials comparing maintenance strategies, and correlation of angulation performance with clinical outcomes.
